# The Harveian Oration

**Published:** 1906-11-03

**Authors:** 


### The Hospital

A Journal of

The Medical Sciences and Hospital Administration.

Vol. XLI.—No. 1,050. SATURDAY, NOVEMBER 3, 1906.

### The Harveian Oration.

The vestigia amongst us form a most interesting study. In our bodies they exist as an appendix, as a pineal body and as a pituitary gland: in our customs they are seen in our clothes, at public dinners; and even in our clothes, as Herbert Spencer pointed out, we still carry evidence of habits which have long become obsolete. The medical profession is not without its vestigia or remnants of a long bygone age, and the College of Physicians, the most conservative body in London, fittingly preserves them and adapts them to a useful modern purpose. In October every year it gathers its Fellows together and professedly in accordance with the directions of William Harvey, its greatest Fellow, it bids them dine in love and harmony on the morrow of the Feast of St. Luke. But the festival is itself long anterior to Harvey, or even St. Luke. In the earlier days the patron Saints of medicine were SS. Cosmas and Damian, who were themselves merely a Christianised form of the great Æsculapius. They lived in complete abstinence and ministered to the wants of all who sought their help, animal as well as human. They exercised their art only for the love of God and for Charity, so that they were known to all men as the moneyless or those who wrought without fee, and were thus symbolical of medicine as a profession and not as a trade. The feast is older even than the patrons. Like all love feasts it goes back to prehistoric times and enshrines a prehistoric idea. It points out that those who partook of common food became fellows or *commensales*, united by a peculiarly intimate bond of union. The idea developed along other lines into the blood brotherhood of many savage tribes. But whether as blood brotherhood or as a modern feast the full acceptation of the rite demanded a victim, who might be a god as in the old pagan rites, an individual, as in the earlier savage times, or an animal as is common amongst the Jews of to-day, who still eat the Passover lamb.

It may well be said that at the Harveian Oration the orator of the year supplies the victim for the feast, and, if so, he carries out the aboriginal idea, for he is carefully selected from the most distinguished of the flock, and he performs his duty most nobly. Professor Osler this year was no exception to the general rule. He took as the subject of his oration, "The Growth of Truth," as illustrated by the history of Harvey's great discovery of the circulation of the blood. He pointed out that all scientific truth was conditioned by the state of knowledge at the time of its announcement. Thus, at the beginning of the seventeenth century, the science of optics and its mechanical appliances had not made it possible for Harvey and his predecessors to imagine the existence of blood corpuscles. Jenner could not, in like manner, have added to his researches on vaccination a discourse on immunity. Sir William Perkin and the chemists made Koch possible; Pasteur gave the conditions which produced Lister; whilst Sir Humphry Davy and others furnished the preliminaries necessary for anæsthesia.

Professor Osler then drew attention to the saddest feature of an inventor's experience the difficulty of obtaining acceptance of a new discovery by men of the same generation. Scarcely a discovery could be named which does not present this phase in its evolution. In a hundred important problems acquisition has by slow stages become latent possession and then there needed but the final touch, the crystal in the saturated solution, to give us conscious possession of the truth. When those stages were ended, there remained the final struggle for general acceptance. Locke's remark that "Truth scarce ever yet carried it by vote anywhere at its first appearance" is borne out by the history of all discoveries of the first rank. The orator recalled an introductory lecture to which he had listened in 1873, the burden of which was "The Finality of Surgery." The lecturer, dwelling on the achievements of the past, concluded that the art had all but reached its limit, little dreaming that within a mile from where he stood the truth for which he and others had been striving, now a conscious possession in the hands of Joseph Lister, would revolutionise it.

The iron yoke of conformity was upon all necks, and in our minds, as in our bodies, the force of habit became irresistible. From our teachers and associates, from our reading, from the social atmosphere about us, we caught the beliefs of the day, and they became part of our nature. In departing from any settled opinion or belief, the variation, the change, the break with custom might come gradually, but the final break was made by some one individual. He often paid dearly for his boldness; and the man who expressed a new idea was very apt to be abused and ill-treated. However eminent a man might become in science, he was very apt to carry with him errors which were in vogue when he was young, errors that darkened his understanding, and made him incapable of accepting even the most obvious truths. It was a great consolation to know that even Harvey came within the range of this law; it was the most human touch in his career.

